# On the prenatal origins of the myeloproliferative neoplasms

**DOI:** 10.17179/excli2025-9181

**Published:** 2026-01-13

**Authors:** Stephen E. Langabeer

**Affiliations:** 1 Cancer Molecular Diagnostics, St. James's Hospital, Dublin, Ireland

## ⁯⁯⁯

The Philadelphia chromosome-negative myeloproliferative neoplasms (MPN) of polycythemia vera (PV), essential thrombocythemia (ET) and primary myelofibrosis (PMF) are hematopoietic stem cell disorders molecularly characterised by on over-production of mature blood cells. MPN are driven by somatically acquired mutations in the *JAK2*, *CALR* and *MPL* genes that result in constitutively activated JAK-STAT signalling and are clinically characterised by a thrombotic tendency and the potential for myelofibrotic or leukemic transformation. MPN are typically adult malignancies.

In a recent review it has been suggested that a prenatal origin of MPN was first established in the years 2021 to 2022 (Chee and Mead, 2024[1]). Although landmark advances in the understanding of MPN clonal architecture were published in these years, cumulative evidence for a prenatal origin derives from several observations prior to these studies. Initial investigations focussed on childhood patients with an early onset of MPN. In an infant with PV and a four-year-old with ET, both harbouring the MPN-specific *JAK2 *V617F mutation, backtracking revealed the presence of this mutation in the neonatal blood spots (NBS) of both patients (Kelly et al., 2008[4]; Langabeer et al., 2013[5]). This approach was later used in a study of adult MPN in which the *JAK2 *V617F mutation was detected in the NBS of a 34-year-old with PV (Sousos et al., 2022[6]). In an intriguing case, an AML patient developed a *JAK2 *V617F donor-derived AML clinically and molecularly reminiscent of the acute transformation of an MPN: analysis of the product used for allogeneic stem cell transplantation revealed the presence of the *JAK2 *V617F in the cord blood stem cells (Hirsch et al., 2016[3]). Further supporting this observation that MPN lesions can be acquired in the prenatal period came the striking observations of firstly concordant ET (Valdés-Mas et al., 2016[7]) and subsequently concordant PMF (Sousos et al., 2022[6]) in monozygotic twins. Both sets of twins had protracted latencies to MPN presentation, and both sets carried the same common *CALR* deletion mutation. Whole genome sequencing (WGS) demonstrated a monoclonal origin of each MPN with trans-placental transfer the most likely mechanism of spread from one twin to the other.

Reconstruction of the clonal architecture and phylogeny of hematopoiesis in *JAK2 *V617F-mutated MPN patients has come from WGS of colonies derived from single hematopoietic stem cells (HSCs). The unique pattern of somatic alterations of each colony can be used to infer the divisional history and relatedness of the HSCs enabling a calculation of when the *JAK2 *V617F mutation was first acquired (Van Egeren et al., 2021[8]; Williams et al., 2022[9]). Both efforts concluded that the *JAK2 *V617F was acquired several decades prior to MPN diagnosis and with estimates varying from a very early childhood to an *in-utero* acquisition. Given the complexity of studying HSCs, mathematical modelling and statistical inference might be used to determine time of disease initiation and prospective dynamics. Such an approach has estimated that *CALR* mutations are unlikely to occur prenatally, the acquisition time for the *JAK2 *V617F is shorter than that of *CALR* mutations, and that *CALR* malignant clones possess a higher proliferative advantage (Hermange et al., 2022[2]). Perhaps somewhat provocatively, this mathematical modelling approach may be used to infer MPN development in relation to early screening strategies suggesting the optimal age for *JAK2 *V617F and *CALR* mutation screening is at 30 and 35 years of age respectively.

Relatively simple studies are clearly warranted on the detection of driver mutations in NBS of adults with sporadic MPN and, together with the power of WGS and single-cell sequencing approaches, will provide further insight into understanding the factors affecting latency and the dynamics of MPN development. Looking to the future, a major aim would be to screen normal, healthy individuals for early signs of MPN driver mutations thus opening a window for therapeutic intervention.

## Declaration

### Acknowledgements

None

### Conflict of interest

The author declares no competing interests.

### Funding

The author declares that no funds, grants or other support were received regarding preparation of this manuscript.

### Artificial Intelligence (AI) - assisted technology

The author declares that no artificial intelligence (AI)-assisted technologies (such as Large Language Models [LLMs], chatbots, or image creators) have been used in the production of the submission.

## References

[1] Chee A, Mead AJ (2024). Molecular profiling in MPN: who should have it and why?. Hematology Am Soc Hematol Educ program.

[2] Hermange G, Rakotonirainy A, Bentriou M, Tisserand A, El-Khoury M, Girodon F (2022). Inferring the initiation and development of myeloproliferative neoplasms. Proc Natl Acad Sci USA.

[3] Hirsch P, Mamez AC, Belhocine R, Lapusan S, Tang R, Suner L (2016). Clonal history of a cord blood donor cell leukemia with prenatal somatic JAK2 V617F mutation. Leukemia.

[4] Kelly K, McMahon C, Langabeer S, Eliwan H, O’Marcaigh A, Smith OP (2008). Congenital polycythemia vera: where does the genotype-phenotype diversity end?. Blood.

[5] Langabeer SE, Haslam K, McMahon C (2013). A prenatal origin of childhood essential thrombocythaemia. Br J Haematol.

[6] Sousos N, Ní Leathlobhair M, Simoglou Karali C, Louka E, Bienz N, Royston D (2022). In utero origin of myelofibrosis presenting in adult monozygotic twins. Nat Med.

[7] Valdés-Mas R, Guitiérrez-Abril J, Pitiot AS, Santamaría I, Puente DA, Muñiz Lobato S (2016). Transplacental transfer of essential thrombocythemia in monozygotic twins. Blood.

[8] Van Egeren D, Escabi J, Nguyen M, Liu S, Reilly CR, Patel S (2021). Reconstructing the lineage histories and differentiation trajectories of individual cancer cells in myeloproliferative neoplasms. Cell Stem Cell.

[9] Williams N, Lee J, Mitchell E, Moore L, Baxter EJ, Hewinson J (2022). Life histories of myeloproliferative neoplasms inferred from phylogenies. Nature.

